# A Multicellular In Vitro Model of the Human Intestine with Immunocompetent Features Highlights Host‐Pathogen Interactions During Early *Salmonella* Typhimurium Infection

**DOI:** 10.1002/advs.202411233

**Published:** 2025-01-14

**Authors:** Spyridon Damigos, Aylin Caliskan, Gisela Wajant, Sara Giddins, Adriana Moldovan, Sabine Kuhn, Evelyn Putz, Thomas Dandekar, Thomas Rudel, Alexander J. Westermann, Daniela Zdzieblo

**Affiliations:** ^1^ Department for Functional Materials in Medicine and Dentistry University Hospital Würzburg Würzburg Germany; ^2^ Department of Bioinformatics Biocenter University of Würzburg Würzburg Germany; ^3^ Department of Microbiology Biocenter University of Würzburg Würzburg Germany; ^4^ Institute of Clinical Transfusion Medicine and Hemotherapy University of Wuerzburg Wuerzburg Germany; ^5^ Helmholtz‐Institute for RNA‐based Infection Research (HIRI) Helmholtz Centre for Infection Research (HZI) Würzburg Germany; ^6^ Translational Center for Regenerative Therapies (TLC‐RT) Fraunhofer Institute for Silicate Research (ISC) 97070 Würzburg Germany

**Keywords:** host‐pathogen interaction, human in vitro infection models, immunocompetent models, intestinal tissue models, microfold cells, organoids, *Salmonella enterica* serovar Typhimurium

## Abstract

Studying the molecular basis of intestinal infections caused by enteric pathogens at the tissue level is challenging, because most human intestinal infection models have limitations, and results obtained from animals may not reflect the human situation. Infections with *Salmonella enterica* serovar Typhimurium (STm) have different outcomes between organisms. 3D tissue modeling of primary human material provides alternatives to animal experimentation, but epithelial co‐culture with immune cells remains difficult. Macrophages, for instance, contribute to the immunocompetence of native tissue, yet their incorporation into human epithelial tissue models is challenging. A 3D immunocompetent tissue model of the human small intestine based on decellularized submucosa enriched with monocyte‐derived macrophages (MDM) is established. The multicellular model recapitulated in vivo‐like cellular diversity, especially the induction of GP2 positive microfold (M) cells. Infection studies with STm reveal that the pathogen physically interacts with these M‐like cells. MDMs show trans‐epithelial migration and phagocytosed STm within the model and the levels of inflammatory cytokines are induced upon STm infection. Infected epithelial cells are shed into the supernatant, potentially reflecting an intracellular reservoir of invasion‐primed STm. Together, the 3D model of the human intestinal epithelium bears potential as an alternative to animals to identify human‐specific processes underlying enteric bacterial infections.

## Graphical abstract

In this work, a human primary cell‐based immunocompetent model is established to investigate early responses to STm infection. Intestinal fibroblasts and monocyte‐derived macrophages are embedded in decellularized small intestinal submucosa (SIS) from domestic pigs, followed by the development of an enteroid monolayer. The generated model highlights human responses and can further serve as a platform in enteric pathogen research.

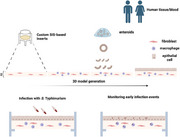

## 1 Introduction

Non‐typhoidal *Salmonella* (NTS) represents a major global cause of food poisoning, with STm being one of the most prevalent strains, due to the extensive range of dietary products through which it can be transmitted.^[^ ^1^, ^2^ ^]^ STm is pathogenic to a broad range of host species and typically induces self‐limiting gastroenteritis in immunocompetent human individuals.^[^ ^3^ ^]^ In immunocompromised individuals, invasive non‐typhoidal salmonellosis (iNTS) manifests as bacteremia with systemic dissemination of STm and chronic shedding to the environment.^[^ ^4^, ^5^ ^]^ In sub‐Saharan Africa, iNTS‐related morbidity represents a global challenge, while multi‐drug resistant strains indicate a potent hazard.^[^ ^6^, ^7^, ^8^ ^]^ The Centers for Disease Control and Prevention (CDC) estimates ≈1.35 million illnesses due to NTS in the US each year, leading to thousands of hospitalizations and hundreds of deaths.^[^ ^9^ ^]^ So far, STm has been extensively studied using mice as a translational model of typhoid fever in humans and the extent to which these findings can be extrapolated to the human host seems limited.^[^ ^8^, ^10^ ^]^ While mouse models provide valuable insights, they do not fully capture the complexities of human‐specific STm infection. To better understand host‐pathogen interactions in humans that contribute to the varying outcomes of STm infections, there is a need for advanced human‐centric models. These models should aim to reconstitute native human tissues in cell culture systems as close as possible, allowing for a more accurate representation of the infection process and enhancing our understanding of how different factors influence the disease in humans.

3D tissue and organoid models have been used to study bacterial infection in vitro reviewed in.^[^ ^11^ ^]^ For example, Transwell‐based systems have been established based on enteroid layers co‐cultured with immune cells and used for bacterial colonization.^[^ ^12^, ^13^ ^]^ However, these setups utilized synthetic matrices,^[^ ^12^, ^13^ ^]^ ignoring the native 3D interactions with the extracellular matrix (ECM) or were based on immortalized cell lines,^[^ ^13^ ^]^ representing an artificial surrogate of the native primary tissue. In our own studies, we have previously established Transwell‐like systems based on the small intestinal submucosa (SIS) from domestic pigs.^[^ ^14^, ^15^ ^]^ SIS serves as a biological scaffold that facilitates the 3D culture of mesenchymal cells while preserving native ECM components of the basal lamina promoting the development of primary adult enteroid layers.^[^ ^16^, ^17^, ^18^ ^]^ In one example, the SIS was seeded with primary human epithelial cells and infected with STm.^[^ ^14^ ^]^ However, due to the lack of an immune compartment in this host model, systemic responses to the infection could not be traced. In another project, we developed a SIS‐based Transwell‐like model that did not only comprise an epithelial compartment, but also a vascular immune cell component comprised of endothelial cells, monocytes, and natural killer cells.^[^ ^15^ ^]^ Infection of this model with STm followed by transcriptomic analysis of the individual cell types allowed us to delineate localized from systemic host responses to infection. However, this co‐culture model was based on human cancer‐derived epithelial cells, and it remained unclear to what extent the obtained findings reflected the native response of primary human tissue. Taken together, the generation of immunocompetent tissue models remains challenging, and immunocompetent systems based on biological ECM and primary human material comprise emerging platforms for in vitro infection studies.

Macrophages are key players in innate and adaptive immunity but also play a major role in gut development and homeostasis.^[^ ^19^ ^]^ For example, macrophages contribute to homeostatic processes such as remodeling of the ECM by secreting matrix metalloproteases (MMP).^[^ ^20^ ^]^ Matrix remodeling has been associated with polarization toward the M2‐like phenotype following the M1‐M2 paradigm.^[^ ^21^ ^]^ In addition, co‐culturing with induced pluripotent stem cell‐derived macrophages guided intestinal differentiation toward the native fetal gut.^[^ ^22^ ^]^ Importantly, macrophages seem to have divergent roles in the host response to STm infection between mice and humans.^[^ ^23^ ^]^ For example, *ex vivo*‐infected primary human macrophages were highly effective in eliminating STm, whereas mouse macrophages maintained numerous viable intracellular bacteria over 24 h of infection.

Besides macrophages, a paradigm example to illustrate our limited knowledge of the virulence strategies of even long‐standing model pathogens relates to the presumed role of microfold (M) cells in STm infection.^[^ ^24^ ^]^ M cells reside within the follicle‐associated epithelium of the gut‐associated lymphoid tissue and represent a preferred entry site of STm into the murine intestine.^[^ ^25^ ^]^ Salmonella interacts with M cells through the FimH on its type I fimbriae, which binds with high affinity to the glycoprotein 2 (GP2) receptor expressed on the M cell surface.^[^ ^26^ ^]^ In contrast to mice, the relevance of M cells for human infections remains partially enigmatic due to the difficulties in reconstituting this cell type in vitro.^[^ ^27^, ^28^, ^29^, ^30^ ^]^ While M cells are considered to serve as an entry point of typhoidal *Salmonella* in humans,^[^ ^31^ ^]^ a previous study has further supported the interaction of STm with M cells on human enteroid‐derived monolayers.^[^ ^32^ ^]^

Here, we use 3D human primary intestinal tissue modeling to address some of the above STm virulence aspects in a human situation. Following up previous attempts to approximate the native tissue microenvironment during STm infection, we generated an immunocompetent primary tissue model of the human small intestine based on SIS, with embedded monocyte‐derived macrophages (MDMs).^[^ ^14^, ^15^, ^16^ ^]^ Once established, we exposed the model to STm and traced host‐pathogen interaction processes using different imaging, molecular, and functional readouts. Taken together, this work presents an advanced 3D in vitro platform for enteric infection research.

## 2 Results

### 2.1 Establishment and Characterization of an Immunocompetent Multicellular Small Intestinal Tissue Model

**Figure 1:**
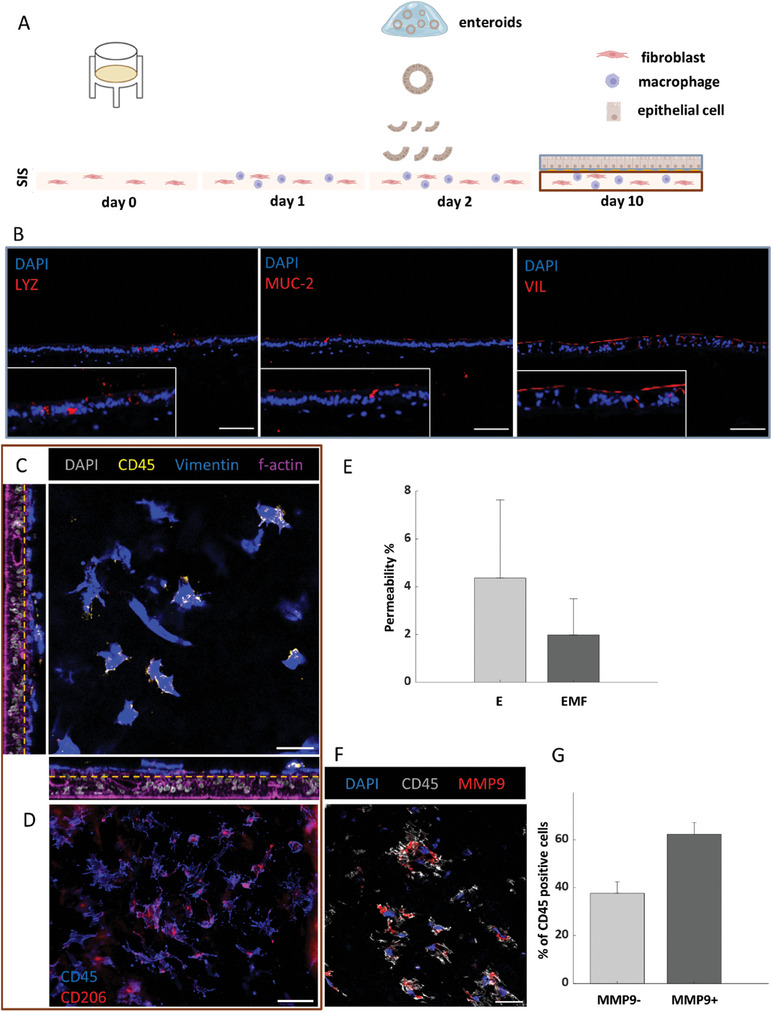
Establishment and characterization of an immunocompetent human intestinal epithelial model. A) Illustration of the experimental steps followed during model assembly. Created with Biorender.com B) Representative microscope images of IHC‐stained tissue sections highlighting cells positive for the Paneth cell marker LYZ (left panel) the Goblet cell marker MUC‐2 (middle panel), and the enterocyte marker VIL (right panel). Cell type‐specific markers are in red. DAPI counterstaining is in blue. Scale bar = 100 µm. (*n* = 3). C) Confocal image depicting 3D subepithelial distribution of fibroblasts (vimentin+, CD45‐) and MDMs (vimentin+, CD45+). Dotted line represents the epithelial boarder. Scale bar = 40 µm. (*n* = 2). D) Epifluorescence image showing CD206 expression by CD45‐positive cells within the fibroblast/macrophage layer. Scale bar = 100 µm. (*n* = 3). E) FITC‐dextran assay to quantify the permeability of the epithelial monolayer and the co‐culture model (E: epithelium; EMF: epithelium‐MDM‐fibroblast). Bars and error bars refer to the mean and standard deviation. (*n* = 3). F) Immunofluorescence staining of MMP9 as a proxy for MDM‐mediated matrix remodeling. G) Quantification of MMP9‐negative and MMP9‐positive MDM populations, based on immunofluorescence data as shown in panel F. Bars and error bars relate to the mean and standard deviation. (*n* = 3). IHC: immunohistochemistry, LYZ: lysozyme, MUC‐2: mucin 2, VIL: villin‐1, MDMs: monocyte‐derived macrophages, MMP9: matrix metalloproteinase 9.

The assembly of the multicellular in vitro model of the human small intestine with immunocompetent features was achieved by embedding monocyte‐derived macrophages (MDMs) together with primary intestinal fibroblasts in the small intestinal submucosa (SIS) matrix, followed by the establishment of an intestinal enteroid layer at the apical side of the matrix (**Figure** **1**A). Immunohistochemical analyses of tissue sections revealed the presence of the intestinal epithelial markers lysozyme and mucin‐2 in the epithelial layer, indicative of secretory epithelial cell types with antimicrobial properties (Figure 1B). The presence of mucin‐2 and villin‐1 was supported by western blotting (Figure S1A, Supporting Information). Fibroblasts and MDMs displayed a 3D distribution within the biological scaffold (Figure 1C), with the expression of CD206 indicating the presence of M2‐like MDMs in the tissue model (Figure 1D).

The integrity of the model was assessed using a FITC‐dextran permeability assay and compared to that of monoculture layers (Figure 1E). The multicellular model was associated with a lower average permeability, although no statistical significance was reached. Immunocytochemical analyses revealed that over 60% of the MDMs expressed MMP9 (Figure 1F,G), in accordance with the matrix remodeling potential in tissue macrophages.^[^ ^33^ ^]^ Finally, migration of the MDMs was observed in the tissue model via live cell imaging (Video S1, Supporting Information), potentiating our previous observations on matrix remodeling.

### 2.2 Transcriptomic Comparison of Different Co‐Culture Models

**Figure 2:**
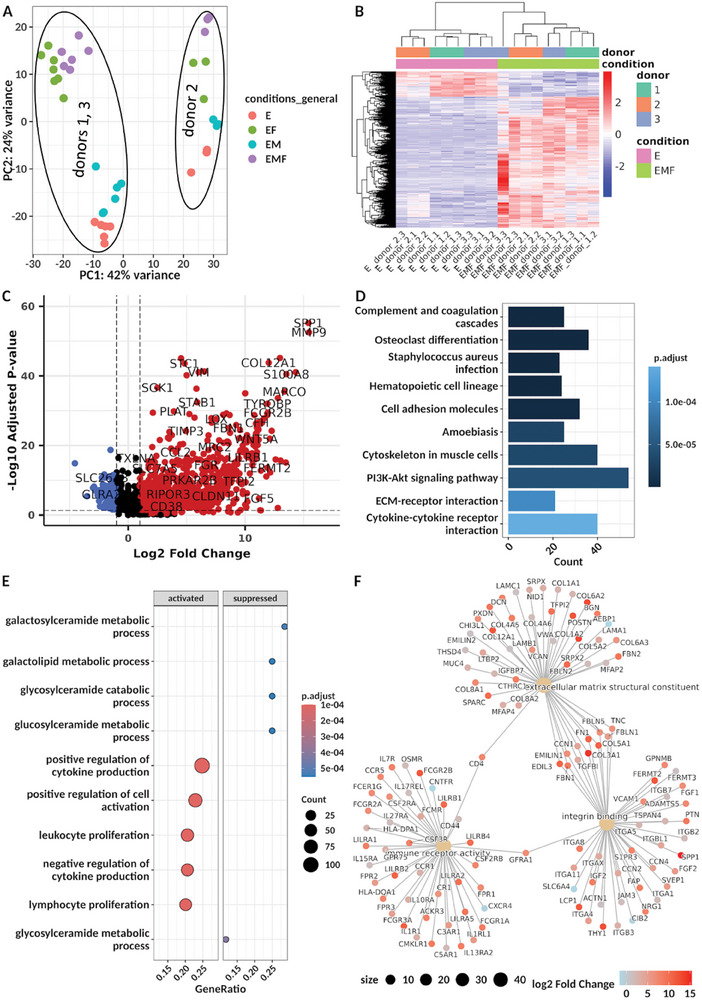
RNA‐seq analysis reveals differences in gene expression between the epithelium only and the epithelium‐MDM‐fibroblast model. A) Principal component analysis of the different RNA samples (E, epithelium; EF, epithelium‐fibroblast; EM, epithelium‐MDM; EMF, epithelium‐MDM‐fibroblast) obtained from three different donors. B, C) Heat map B) and volcano plot C) visualizing the gene expression differences between E and EMF models. D–F) Functional analyses of the transcriptome data. The 10 top‐ranked pathways from a KEGG enrichment analysis D), the 5 top‐ranked activated and suppressed biological processes according to GSEA E), and a CNET plot visualizing the three most significant Gene Ontology molecular functions F), all based on genes differentially abundant between EMF and E models. Genes enriched or depleted in the EMF co‐culture model in comparison to the epithelial monoculture are in red or blue, respectively, throughout this figure.

To complement the above‐targeted characterization, the tissue model was next subjected to global transcriptomic analysis using bulk mRNA sequencing. In particular, we aimed to compare the gene expression of the fully assembled model (epithelium, MDMs, fibroblasts; EMF) with its cellular constituents, namely epithelium only (E), co‐culture of epithelial cells and fibroblasts (EF), and of epithelial cells and MDMs (EM), in order to identify gene expression signatures of specific cell type compositions. Principal component analysis (PCA) segregated the samples primarily by donor, specifically delineating donor #2 from the two remaining donors (**Figure** **2**A,B). Reassuringly, however, there was an underlying clustering based on cell type composition. Accordingly, differential expression analysis identified sets of genes that were enriched or depleted in the complete EMF model relative to the pure epithelial control (Figure 2C). For example, genes involved in ECM organization were prominently enriched in the EMF model. Furthermore, by deconvolution transcriptome analysis (see Methods), we found that fibroblasts were characterized by type‐I and type‐III collagen expression as well as the gene of the metalloprotease inhibitor TIMP3 (Figure S2, Supporting Information). On the other hand, MDMs were characterized by abundant levels of *MMP9* mRNA, reflecting the corresponding protein levels (Figure 1F,G), while epithelial co‐culture with fibroblasts resulted in a significant increase in *MMP2* expression (Table S3, Supporting Information). Both MMP2 and MMP9 can cleave type‐IV and –V collagens, together with elastin and vitronectin, which have been previously characterized in the SIS composition.^[^ ^17^, ^34^ ^]^ Fibroblasts further contributed to the upregulation of *WNT5A, WNT5B*, and *CLDN11* (Figure S2, Supporting Information), suggesting an involvement in epithelial development and maintenance of barrier integrity.^[^ ^35^, ^36^, ^37^ ^]^ Addition of MDMs to the epithelial cell culture led to an induction of *MARCO*, encoding a non‐opsonic phagocytotic receptor also associated with peritoneal, splenic, and alveolar macrophages.^[^ ^38^, ^39^ ^]^ KEGG pathway, gene ontology, and gene set enrichment analyses revealed biological processes related to immunity and the ECM to be differentially represented between EMF and E models (Figure 2D–F). Altogether, this transcriptomic analysis therefore further supports our intestinal EMF model to recapitulate key properties of the in vivo situation.

### 2.3 STm Interacts with M‐Like Cells Early after Infection of the Epithelial‐MDM‐Fibroblast Co‐Culture Model

**Figure 3:**
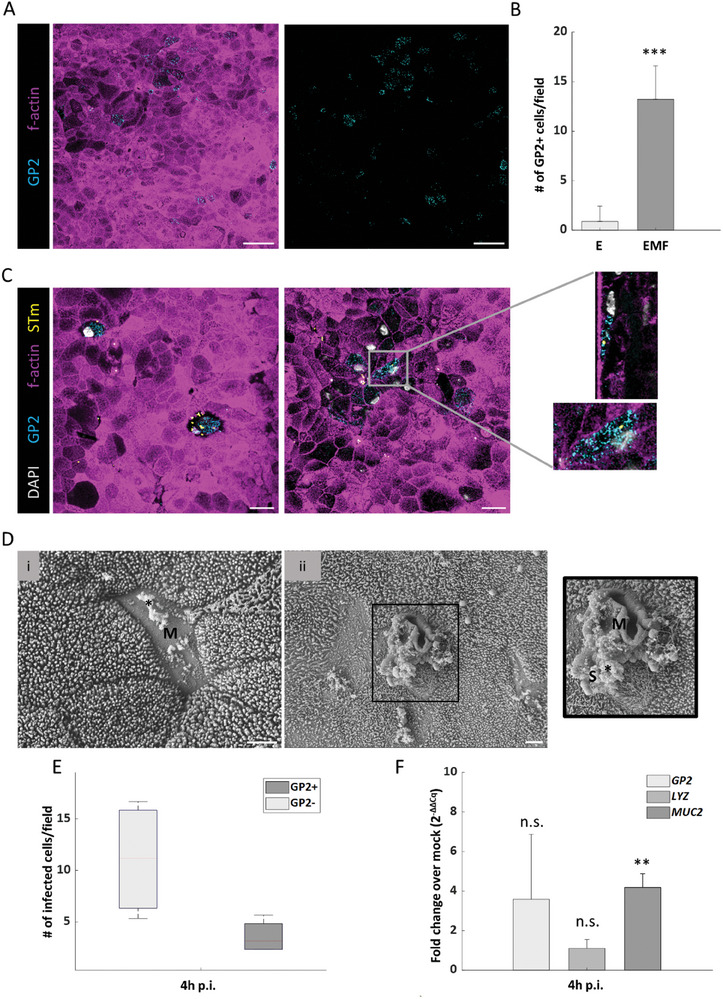
Identification and functional analysis of M‐like cells in the EMF model. A) Immunofluorescence microscopy indicates that GP2 is expressed by a sub‐population of epithelial cells in the EMF model. Scale bar = 40 µm. (*n* = 4). B) Bar plot shows mean counts for GP2‐positivecells in E and EMF models based on immunofluorescence analysis as depicted in panel A. Error bars indicate the standard deviation from the mean. (*n* = 3). (***) *p* ≤ 0.001, Student's *t*‐test. C) Infection of EMF models with STm (MOI 10; 4 h p.i.) followed by fluorescence microscopy. GP2‐positive cells are in blue, STm in yellow, and actin staining is shown in magenta. Left panel: confocal image showing STm invasion into GP2‐positive cells. Right panel: intracellular localization of STm in GP2‐expressing cells. Scale bar = 20 µm. (*n* = 4). D) Visualization of M‐like cells via SEM of (i) uninfected (left) and (ii) STm‐infected (right, 4 h p.i.) EMF models. *: mucus; M: M‐like cell; S: S. Typhimurium. Scale bars = 2 µm. (*n* = 1). E) Quantification of counts of infected cells per confocal microscopy image field. Red line represents the median and whiskers denote the minimum and maximum values (*n* = 4). F) qRT‐PCR‐based analysis of the host response to STm infection at 4 h p.i. Bars represent the mean and error bars the standard deviation from the mean. (*n* = 3). (**) *p* ≤ 0.01; Student's *t*‐test, n.s.: non‐significant. GP2: glycoprotein 2, E: epithelium, EMF: epithelium‐MDM‐fibroblast, SEM: scanning electron microscopy, MDMs: monocyte‐derived macrophages.

Given that our RNA‐seq analysis hinted at the presence of M‐like cells in our EMF co‐culture model (Figure S3B, Supporting Information), we followed up on this possibility on the protein expression level. Albeit, deconvolution analysis could detect any significant difference between the E and the EMF models, immunocytochemical analysis supported the expression of the M cell marker GP2 by the epithelial layer of the EMF model (**Figure** **3**A). The number of GP2‐expressing cells increased drastically in the EMF model relative to the epithelial monoculture (Figure 3B). The interaction of GP2 positive cells with latex beads in the EMF model, which was not found in the epithelial monoculture (Figure S3A, Supporting Information), provided further evidence for the presence and activity of M‐like cells in the co‐culture model.^[^ ^28^, ^40^ ^]^ Comparing gene expression of co‐cultures with fibroblasts and macrophages to via qRT‐PCR suggested elevated levels of tumor necrosis factor superfamily (TNSF) members in the presence of MDMs (Figure S3C, Supporting Information). We then hypothesized that the exclusive detection of lymphotoxin‐β (*LTB*) mRNA in the presence of MDMs might imply epithelial maturation to depend on LTB signaling, as previously reported.^[^ ^28^, ^41^ ^]^ Indeed, differentiation of the epithelial monolayer in the presence of the TNFSF member and lymphotoxin‐β receptor (LTβR) agonist^[^ ^42^ ^]^ LIGHT, resulted in the induction of GP2 within enteroid‐derived cells on the apical surface of the model (Figure S3D, Supporting Information). Lastly, scanning electron microscopy (SEM) indicated the presence of folded cells without microvilli, reminiscent of M cell characteristics (Figure 3D,i).

To functionally address the role of these M‐like cells during enteric infection, we challenged the EMF model with STm (Figure 3C). Notably, SEM revealed individual STm bacteria in close vicinity of cells with M cell morphological characteristics and suggested the presence of amorphous material resembling mucus^[^ ^43^ ^]^ at the surface of these cells (Figure 3D,ii). Confocal microscopy demonstrated that at 4 h after infection, a proportion of the STm‐containing epithelial cells stained positive for GP2 (Figure 3E). Quantification of mRNA levels via qRT‐PCR suggested elevated levels of the *GP2* (albeit insignificant) and *MUC2* transcripts in infected compared to uninfected EMF models (Figure 3F).

### 2.4 MDMs Engulf STm and Mount a Pronounced Innate Immune Response

**Figure 4:**
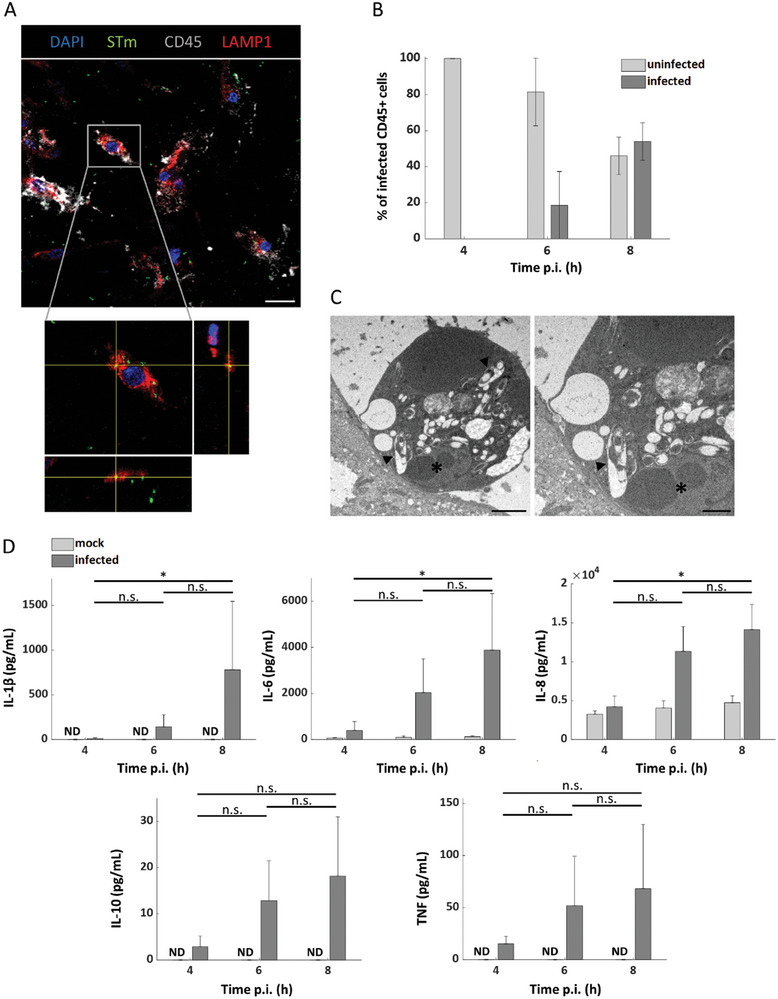
Characterization of macrophages within the EMF model during STm infection. A) Confocal image showing co‐localization of STm and LAMP1 signal on MDMs (CD45+) at 8 h p.i. Magnified inset highlights the intracellular localization of individual STm bacteria in a vesicular compartment of an infected MDM. Scale bar = 20 µm, (*n* = 3). B) Bar plot shows the mean percentages of infected (dark gray) and uninfected (light gray) MDMs over a time‐course of STm infection, with error bars representing the standard deviation from the mean, (*n* = 3). C) Electron micrographs depict the interaction of MDMs with the intestinal epithelium during STm infection. Left panel shows an infected MDM with a single STm contained in a vacuolar compartment at 4 h p.i. (arrow). The nucleus is labeled (*). Scale bars = 2 µm (low magnification; left) or 1 µm (high magnification; right), (*n* = 3). D) Levels of inflammatory cytokines measured in the supernatants of the EMF model by ELISA. Bars and error bars represent the mean and standard deviation, (*n* = 4). (*) *p* ≤ 0.05; Kruskal‐Wallis test with Dunn's post‐hoc test, n.s.: non‐significant. LAMP1: lysosome‐associated membrane protein 1, MDMs: monocyte‐derived macrophages, ND: non‐detected.

Near the epithelial layer, intestinal phagocytes can engulf luminal bacteria.^[^ ^44^ ^]^ To test for a potential trans‐epithelial phagocytosis of STm by MDMs in our model, we applied different immunostaining techniques. We observed STm co‐localization with the lysosomal marker LAMP1 inside MDMs (**Figure** **4**A). The incidence of MDMs harboring intracellular bacteria was rare at early time points (4 and 6 h p.i.), yet at 8 h p.i. about half of the MDMs in the model contained STm (Figure 4B). Uptake of STm by MDMs was corroborated by transmission electron microscopy (TEM) (Figure 4C). Rare instances of luminal compartmentalization and phagocytosis were noted at 8 h p.i., indicating trans‐epithelial migration of MDMs and pathogen clearance within the lumen (Figure S4A,B, Supporting Information). The tissue model mounted a strong inflammatory response to STm infection, as evidenced by the secretion of IL‐1β and TNF, while the anti‐inflammatory cytokine IL‐10 was also detected (Figure 4D). Increased levels of IL‐6 and IL‐8 were also observed in the supernatants after infection with STm. Collectively, these findings suggest that MDMs maintain their phagocytic activity in the EMF model accompanied by a distinct cytokine secretion during STm infection.

### 2.5 Apical Extrusion of STm‐Infected Epithelial Cells in the EMF Model

**Figure 5:**
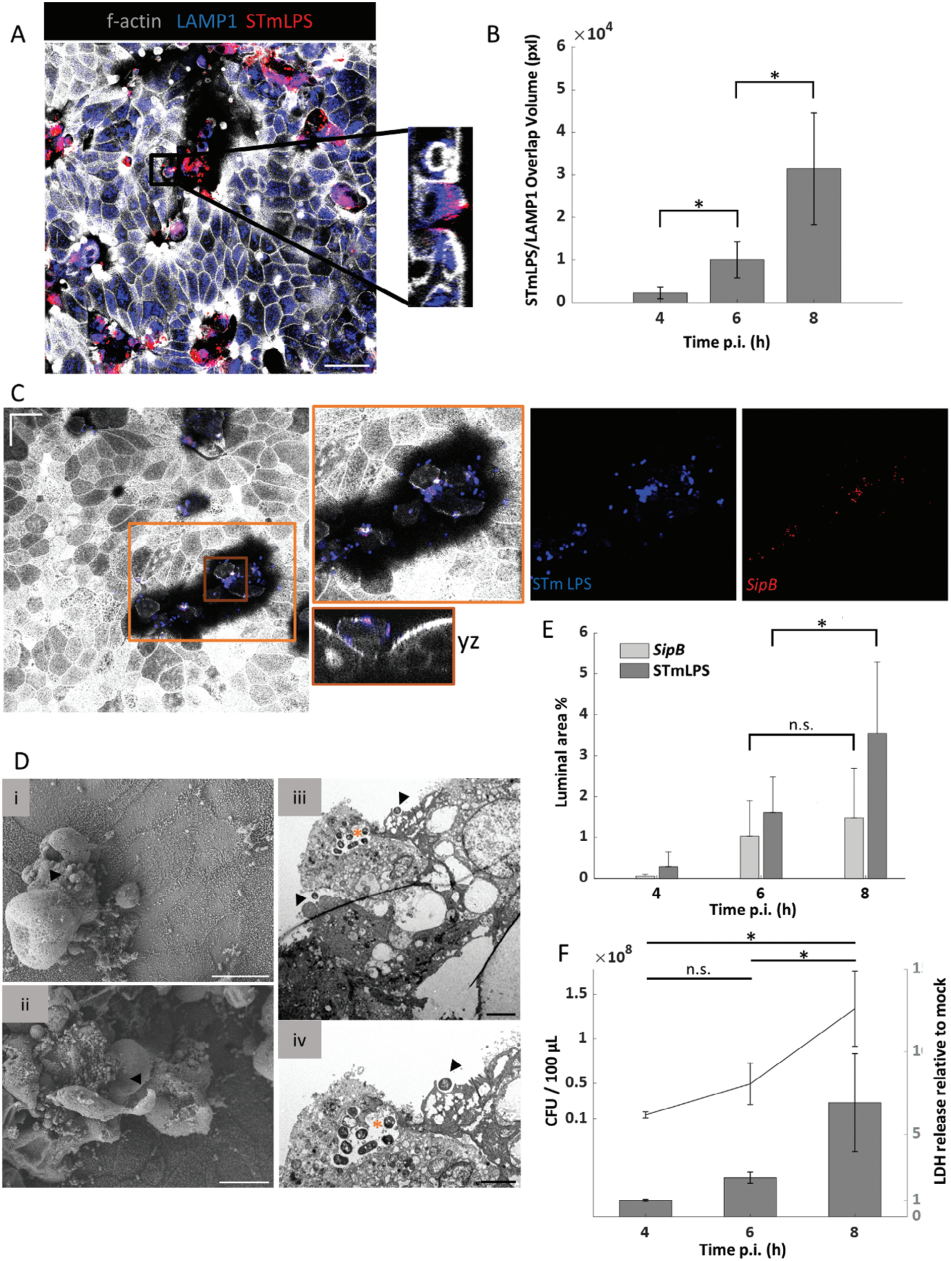
Intraepithelial STm within the EMF model localizes to SCVs and their host cells are expelled from the epithelium at later infection stages. A) Immunofluorescence image shows LAMP1 signal co‐localized with STmLPS at 6 h p.i. XZ magnification shows an epithelial cell with vacuolar STm, with disrupted polarity and cell‐cell junctions. Scale bar = 40 µm, (*n* = 3). B) Bar plot displays the mean overlap volume of LAMP1 with STmLPS over the time‐course of infection. Error bars represent the standard deviation from the mean, (*n* = 3). (*) *p* ≤ 0.05; Friedman's test with Dunn's post‐hoc test C) Immunofluorescence combined with HCR‐RNA FISH. The confocal image shows the localization of STm and the FISH signal derived from the bacterial *sipB* mRNA as a proxy for an STm sub‐population that induces its invasion machinery. Scale bar = 20 µm, (*n* = 3). D) Ultrastructural analysis of STm‐infected epithelial cells. i) SEM image of an extruded epithelial cell at 4 h p.i. The arrowhead indicates reemerging STm. Scale bar = 10 µm. ii) Region with an expelled epithelial cell cluster at 8 h p.i. The arrowhead points at a released, flagellated STm bacterium. Scale bar = 10 µm, (*n* = 1). iii) TEM image shows the extrusion of an epithelial cell with disrupted SCV (*) at 4 h p.i. Scale bar = 2 µm. iv) Magnification of iii. highlighting the disrupted SCV (*), while STm induced ruffling of an adjacent cell (arrowhead), probably initiating a second round of infection. Scale bar = 1 µm, (*n* = 3). E) Bar plot shows the mean relative luminal area stained for *sipB* or STmLPS, respectively, and error bars represent the standard deviation, (*n* = 3). (*) *p* ≤ 0.05; ANOVA with Tukey's post‐hoc test, n.s.: non‐significant. F) Plot showing STm CFU counts (line) in the apical compartment of the model and release of lactate dehydrogenase (LDH; relative to mock‐infected controls; bars) as a proxy for host cytotoxicity at distinct stages of STm infection. Data refer to the mean and standard deviation. (*) *p* ≤ 0.05; n.s.: non‐significant; ANOVA with Tukey's post‐hoc test, n.s.: non‐significant. LAMP1: lysosomal‐associated membrane protein 1, STmLPS: *S*. Typhimurium lipopolysaccharide, *sipB*: mRNA for the *Salmonella* invasion protein B, SCV: *Salmonella*‐containing vacuole, SEM: scanning electron microscopy, TEM: transmission electron microscopy.

During gut homeostasis, epithelial cells are constantly shed into the lumen as a prerequisite for turnover of the intestinal epithelium. Some intracellular bacterial pathogens, including STm, hijack this process and induce host cell extrusion to promote their fecal shedding or the infection of bystander cells.^[^ ^45^, ^46^ ^]^ Subcellular localization studies revealed the responsible STm bacteria to escape from the modified phagosome known as the *Salmonella*‐containing vacuole (SCV),^[^ ^47^ ^]^ but to instead hyperreplicate in the cytosol.^[^ ^45^, ^48^ ^]^ In our EMF model, immunofluorescence analysis revealed a progressive shedding of infected epithelial cells from the epithelial layer at later stages of infection (Figure S5, Supporting Information). However, inside these epithelial cells, STm co‐localized with LAMP1, indicating the maturation of the SCV in intestinal epithelial cells (**Figures** **5**A and 4B). In contrast, the occurrence of cytosolic STm could not be accurately quantified, as LAMP1 is associated with late SCVs.

To address whether these intraepithelial STm are primed for invasion of uninfected bystander cells as previously suggested,^[^ ^45^ ^]^ we established RNA fluorescence in situ hybridization (FISH) for the mRNA encoding the invasion effector SipB. Combination with immunofluorescence against lipopolysaccharide (LPS), an abundant component of the Gram‐negative cell envelope, suggested an invasive intraepithelial STm population to reemerge ≈6 h p.i. (Figure 5C,E). These invasion‐primed STm might account for the observed increase in bacterial CFU counts and be responsible for the induced epithelial damage occurring at 8 h p.i, as indicated by the release of lactate dehydrogenase (LDH). (Figure 5F). Supporting this model, ultrastructural analysis captured the extrusion of infected epithelial cells, disruption of the SCV, and the subsequent release of bacteria from the expelled cells into the supernatant (Figure 5D). At late infection stages (6 and 8 h p.i.), extruded cells accumulated in clusters, generating characteristic 3D structures of localized infection niches (Figure S5, Supporting Information). Altogether, this initial characterization of our immunocompetent epithelial tissue model proposes it as an amenable alternative to animal infection experiments to improve our understanding of the virulence strategies employed by human enteropathogens.

## 3 Discussion

Primary tissue culture models emerge as attractive alternatives to animal models in infection research.^[^ ^11^ ^]^ Whereas previous Transwell‐based systems of primary enteroid‐derived epithelial cells co‐cultured with MDMs relied on synthetic materials,^[^ ^12^ ^]^ in our model the cells were seeded onto a biological and organ‐specific matrix. Our model was developed using pig‐derived SIS due to its accessibility and similarity to the human intestine;^[^ ^49^ ^]^ however, future studies could evaluate the setup with human‐derived SIS. Transcriptomic and immunocytochemical findings further highlighted ECM remodeling processes in the biological matrix, which would not be supported by unmodified, synthetic matrices. SIS supported as a material the co‐culture of MDMs and intestinal fibroblasts together with a primary enteroid layer, in a Transwell‐like setting, modeling the epithelial/subepithelial tissue structure. Previous work using an SIS‐based compartmentalized system,^[^ ^15^ ^]^ which included circulating monocytes, lacked evidence of direct subepithelial interaction between the pathogen and immune cells.^[^ ^15^ ^]^ In our improved model, we developed cell culture methods to embed and maintain MDMs within a 3D scaffold, enabling physical interactions with the pathogen during various early infection stages. Furthermore, our model serves as a platform for investigating tissue‐resident immune cell populations and epithelial development, benefiting significantly from its reliance on primary cell composition. On the other hand, SIS has several limitations, including batch‐to‐batch and microtopographical variations.

Using the well‐characterized STm as a model pathogen, we demonstrated the suitability of our tissue model to recapitulate bacterial virulence and host response strategies in vitro. For example, STm interacted with M‐like cells—a process rarely observed in vitro.^[^ ^32^ ^]^ In previous work, we had demonstrated the presence of M‐like cells in the epithelial monocultures through single‐cell RNA‐seq analysis.^[^ ^14^ ^]^ Similarly in the present work deconvolution analysis supported an M‐like cell phenotype being expressed on the epithelial monocultures. However, the induction of GP2 on the surface of GP2 cells was only detected in the immunocompetent model and further supports the importance of myeloid populations on the maturation of M cells.^[^ ^30^ ^]^ Previous studies have highlighted the role of the TNSF members in the induction of M cells in vitro.^[^ ^27^, ^28^, ^32^, ^40^ ^]^ In this study we introduced LIGHT (TNFSF14) as an alternative TNFSF member which could induce GP2 on the epithelial monocultures. LIGHT binds to the LTβR and the herpes virus entry mediator (HVEM).^[^ ^50^ ^]^ While previous studies have addressed the impact LTβR activation on M cell maturation,^[^ ^28^, ^41^ ^]^ the involvement of HVEM in the M cell development should be further investigated.

Moreover, during infection, we observed direct interaction of the MDMs with the enteroid, including luminal trans‐migration. The MDMs then phagocytosed STm—a well‐known process that occurs in vivo. Here we report for the first time a 3D immunocompetent infection model recapitulating early STm infection stages, providing a state‐of‐the‐art platform. This setup could establish a methodology for the investigation of further pathogens and the cross‐talk of the epithelium with the innate immune component during infection. It was further postulated that luminal colonization of STm leads to re‐invasion from the luminal compartment and further infection of bystander cells and to fecal shedding.^[^ ^51^, ^52^, ^53^, ^54^ ^]^ Our data point in a similar direction, as the EMF model expelled infected intestinal epithelial cells with characteristic SCVs toward the lumen. We were not able to quantify the proportion of cytosolic *Salmonella* inside epithelial cells of the EMF model, which would further highlight the compartmentalization of virulent populations in host cells,^[^ ^45^, ^55^ ^]^ due to limitations of our setup, since LAMP1 cannot capture the total vacuolar *Salmonella* population.

Our system also elicited a distinct inflammatory response, aligning with previous in vivo findings on the cytokine release profile during STm infection.^[^ ^56^, ^57^ ^]^ Previous work has shown the capacity of human intestinal epithelial cells to secrete IL‐8 but not IL‐6 and IL‐1.^[^ ^58^ ^]^ Therefore, we could assume that co‐culture with MDMs primed the secretion of IL‐1, IL‐6, and IL‐10 as a response to the pathogen. Another study demonstrated that neutrophil‐derived IL‐1β drives epithelial cell extrusion,^[^ ^59^ ^]^ a process observed during the later stages of infection in the EMF model. Similarly, tissue damage during the infection could be indicated by the production of TNF which could additionally direct the extrusion of epithelial cells, via apoptotic or necroptotic cell death.^[^ ^60^, ^61^ ^]^ Those facts suggest that the epithelial cell extrusion could be further enhanced by paracrine signals from MDMs besides activation of intraepithelial inflammasomes.^[^ ^51^, ^61^, ^62^ ^]^ IL‐10 which was exclusively detected upon infection, is an anti‐inflammatory cytokine that could underline a homeostatic response from MDMs to the acute infection, in order to limit tissue damage.^[^ ^63^, ^64^ ^]^ *Salmonella* infected MDMs are known to secrete IL‐10,^[^ ^65^ ^]^ however its role as a pleiotropic cytokine during infection remains controversial. Treatment with recombinant IL‐10 has shown to pronounce STm replication in MDMs,^[^ ^66^ ^]^ while disruption of the IL‐10 receptor led to impaired phagocytosis.^[^ ^67^ ^]^ Altogether, our immunocompetent model would offer a platform for the better understanding and further characterization of the cytokine milieu during early STm infection.

In conclusion, we have generated a human intestinal model with 3D embedded MDMs based on a decellularized biological matrix, which could evaluate a variety of innate immune responses against *Salmonella* infection. Although our presented model was developed under static conditions, implementing dynamic culture conditions or a bioreactor pipeline in the future could enhance the model's longevity. The model's lifetime could be prolonged by co‐colonization with commensal microbiota, which might attenuate *Salmonella* overgrowth in the luminal compartment.^[^ ^68^ ^]^ In addition, it would be interesting to address donor‐to‐donor variations during intestinal infections or compare “healthy” to “diseased” models, such as to those established with inflammatory bowel disease‐derived enteroids. Finally, replacing MDMs with IPSC‐derived macrophages could better reflect the diverse genetic backgrounds of donors, paving the way to personalized applications.

## 4 Experimental Section

### Human Tissue

For the isolation of small intestinal crypts containing intestinal stem cell populations, biopsies of human jejunum were obtained from obese patients that underwent a stomach bypass operation at the surgery unit of the University Hospital Würzburg. The study was approved by the Institutional Ethics Committee on human research of the Julius‐Maximilians University Würzburg (approval numbers 182/10 and 280/18sc). Informed written consent was obtained before surgery and data analysis was done anonymously according to the principles expressed in the “Declaration of Helsinki”.

### Enteroid Derivation and Culture

Small intestinal enteroids were derived from non‐inflamed, ‐malignous adult duodenum as previously described.^[^ ^14^, ^16^, ^69^, ^70^ ^]^ Enteroids were fragmented by incubation in TrypLE Express at 37 °C for 4–5 min and passaged by encapsulation in Matrigel domes seeded on pre‐warmed 6‐well tissue culture plates, followed by 15‐minute incubation at 37 °C, 5% CO_2_ to allow cross‐linking. Cells were supplemented with enteroid growth medium consisted of 75% L‐WRN conditioned medium (ATCC) and 25% Advanced DMEM F12 (Gibco) supplemented with, 1× N2‐Supplement (Gibco), 1× B27‐Supplement without vitamin A (Gibco), 10 mm HEPES (Sigma Aldrich), 1x GlutaMax‐I, 1x Anti‐Anti (Gibco), 1 mm N‐Acetylcysteine (Sigma Aldrich), 0.05 µg mL^−1^ EGF (Peprotech), 0.01 µm Leu‐Gastrin (Sigma Aldrich), 10 mm Nicotinamide (Sigma Aldrich), 0.5 µm LY2157299 (CAYMAN Chemical Company), 10 µm SB202190 (Sigma Aldrich), 0.5 µm A83–01 (Tocris), 10 µm Y‐27632 (CAYMAN Chemical Company) and 1 µm JAG‐1 (AnaSpec Inc.). After 2 days in culture, Y‐27632 and JAG‐1 were removed and enteroids were expanded for 2–3 additional days. Cells were routinely tested for mycoplasma contamination.

### Isolation and Culture of Intestinal Fibroblasts

After isolation of crypts, small intestinal submucosal tissue from human biopsies was cut into small pieces using sterile scissors. Tissue pieces were incubated for 40 min at 37 °C on an orbital shaker, in complete DMEM (Gibco,61965‐026) containing 10% FCS (PAN Biotech) and 1x Pen/Strep (Sigma Aldrich) supplemented with 0,3 mg mL^−1^ Dispase (Gibco) and 0,06 mg mL^−1^ TM‐Liberase (Roche). Digestion was interrupted, by adding 2 volumes of complete DMEM. Tissue pieces were strained using a 100 µm strainer with a syringe plunger and washed with 10 mL complete DMEM. Cell suspension was centrifuged and resuspended in complete Fibrolife medium (Lifeline Tecnhologies) with 2% FCS. Cells were used until passage 5. After passage 0, hydroxicortisone was removed from the medium. Cell were routinely tested for mycoplasma contamination. Information on enteroid media constituents is provided on Table S1 (Supporting Information).

### Isolation and Culture of Monocytes

Monocytes were isolated from leukocyte cones that were provided from the Transfusion Medicine Department at the University Hospital of Wuerzburg. Briefly, peripheral blood mononuclear cells (PBMCs) were isolated from buffy coats by Ficoll‐Paque PLUS (Cytiva) density gradient centrifugation. Monocytes were isolated from different donor PBMCs by negative selection using the Human pan‐monocyte isolation kit (Miltenyi). Isolated monocytes were cultured on ultra‐low attachment plates (Corning) in RPMI (Gibco, 61870‐010) supplemented with 10 mm HEPES, Pen‐Strep, and 10% heat‐deactivated FCS, in the presence of 50 ng mL^−1^ M‐CSF (Peprotech) for 6 days at 37 °C, 5% CO_2_. Medium was replaced every 3 days.

### SIS Preparation

Jejunal segments were explanted from domestic pigs according to the German law and institutional guidelines approved by the Ethics Committee of the District of Unterfranken, Würzburg, Germany (approval number 55.2‐2532‐2‐256; 55.2.2‐2532‐2‐1477‐41). Preparation of the SIS was followed as previously described.^[^ ^18^, ^71^ ^]^

### 3D Model Assembly and Culture

SIS scaffolds were mounted onto custom iScript‐like cell crown system and incubated overnight in Advanced DMEM F12 (Gibco) without antibiotics to ensure the absence of contamination as previously mentioned.^[^ ^14^, ^16^ ^]^ On the next day, media was removed and replaced with DMEM (Gibco) supplemented with 10% FCS. Fibroblasts were applied on the apical compartment of the insert, to the density of 25 000 cells per insert. Scaffolds were incubated overnight to allow the incorporation of the fibroblasts. On the next day, media was removed and replaced with DMEM containing 10% FCS, supplemented with 20 ng mL^−1^ M‐CSF, and 50 000 MDMs were applied on the apical compartment. For live imaging and during model establishment, MDMs were incubated in 1 µm CellTracker Deep Red dye (Invitrogen) for 20–30 min at 37 °C. Inserts were incubated overnight. On the following day, the medium was removed and replaced with enteroid growth medium supplemented with Y‐27632 and JAG‐1. The medium on the basolateral compartment was supplemented with 20 ng mL^−1^ M‐CSF to stabilize the polarity and pronounce the survival of the macrophages. Small intestinal enteroid fragments of passages 8–18 were applied on the apical compartment of the insert to the density of 350.000–400.000 cells per model. After 2 days, Y‐27632 and JAG‐1 were removed and models were cultured with enteroid growth medium for 2 additional days. After 4 days of proliferation, media was switched to differentiation medium supplemented with 20 ng mL^−1^ M‐CSF on the basolateral compartment. Differentiation medium was consisted of 25% Wnt‐3A conditioned medium (L‐Wnt‐3A, ATCC) and 75% Advanced DMEM F12 (Gibco) supplemented with, 1× N2‐Supplement (Gibco), 1× B27‐Supplement without vitamin A (Gibco), 10 mm HEPES (Sigma Aldrich), 1x GlutaMax‐I, 1x Anti‐Anti (Gibco), 1 mm N‐Acetylcysteine (Sigma Aldrich), 0.5 µg mL^−1^ R‐Spondin‐1 (Peprotech), 0.1 µg mL^−1^ rec mNoggin (Peprotech), 0.05 µg mL^−1^ EGF (Peprotech), 0.01 µm Leu‐Gastrin (Sigma Aldrich), 0.5 µm LY2157299 (CAYMAN Chemical Company), 10 µm SB202190 (Sigma Aldrich), 0.5 µm A83–01 (Tocris). Models were differentiated for a total period of 4 days. On the third day of differentiation, M‐CSF was removed in order to pre‐prime the macrophages before infection. Information on the model culture media constituents is provided on Table S1 (Supporting Information).

### GP2 Induction on Epithelial Monolayers

Enteroid fragments were harvested and seeded on SIS as discussed above. Enteroid monolayers were expanded for 5–6 days in enteroid growth medium. The medium was replaced after with differentiation medium supplemented with 50 ng mL^−1^ recombinant human LIGHT (Peprotech) and the models were allowed to differentiate for additional 5 days.

### Infection with Salmonella Typhimurium

Infections were conducted using the GFP‐expressing *Salmonella* Typhimurium strain SL1344.^[^ ^72^, ^73^ ^]^ Preparation of the bacterial suspension was conducted as previously described.^[^ ^14^ ^]^ Models were infected with an estimated dose of 250 000 STm, corresponding to a multiplicity of infection (MOI) of 10. Models were incubated for up to 8 h in the absence of gentamicin.

### Latex Bead Assay

Fluorescent red, carboxylate‐modified polystyrene beads (Sigma‐Aldrich) were resuspended in Advanced DMEM F12 (Gibco) and applied on the apical compartment of the models. Models were incubated for 4 h at 37 °C, 5% CO_2_.

### 2D Co‐Culture of Fibroblasts and MDMs

Fibroblasts and MDMs were plated to a density similar to the 3D model as described above, on 48‐well tissue culture plates. Monocultures for each group and co‐cultures were plated in triplicates. 24 h after the addition of MDMs, lysates for RNA isolation were collected per triplicate by washing the wells with TRK lysis buffer (VWR). Lysates were stored at −80 °C for follow‐up analysis.

### FITC‐Dextran Assay

To perform the FITC‐Dextran assay, 0.6 mg of FITC‐Dextran (4 kDa, Sigma) was dissolved in 15 mL of Advanced F12/DMEM, (Gibco) to a final concentration of 0.04 mg mL^−1^ (10 µm). Unconjugated molecules were removed by centrifugation in a 3 kDa Ultra‐15 centrifugal filter device (Amicon) at 4000× *g* for 30 min at RT. The solution retained in the filter was equilibrated with differentiation medium and 300 µL was applied on the apical compartment of the models. Models were incubated at 37 °C, 5% CO_2,_ and aliquots from the basolateral compartment were plated on black bottom 96‐well plates. Fluorescence intensity was read on a Tecan Infinite M200 multimode microplate reader.

### Immunohistochemistry

Paraffin embedding of the models was done in an ascending row of H_2_O, 50% EtOH, 75% EtOH, 90% EtOH, 2‐Propanol, and Xylol with subsequent incubation in liquid paraffin in a Microm STP 120 (ThermoFisher). Cuts of 7 µm thickness, were mounted on poly‐lysine coated slides and dehydrated overnight at 37 °C. Paraffin was melted by incubation at 60 °C for 1 h. Deparaffinization was performed in a descending row of Xylol, 90% EtOH, 75% EtOH, 50% EtOH, and H_2_O. Antigen retrieval was performed in a steam cooker (Braun) for 20 min in 1x citrate buffer pH 6 (Sigma). Sections were next blocked for 20 min in 5% donkey serum diluted in antibody diluent solution (DCS Innovative Diagnostic Systems). Primary antibodies for VIL (Invitrogen, PA5‐29078), MUC‐2 (Abcam, ab76774), LYZ (Invitrogen, PA1‐29680) were diluted 1:200 in antibody diluent solution and incubation was followed overnight at 4 °C. Sections were washed in 1x PBS‐0,5% Tween 20 (PBS‐T) and incubated with Alexa 555 donkey anti‐rabbit (Invitrogen) antibody for 1 h. Samples were washed in PBS‐T and mounted with Fluoromount G containing DAPI (Invitrogen).

### Alcian Blue Histological Staining

Paraffin sections of 7 µm thickness were prepared and deparaffinized as described above. Sections were immerged in 3% acetic acid solution (Roth, ROTIPURAN) at room temperature. Samples were incubated in 1% Alcian Blue solution pH 2 (MORPHISTO) for 30 min at room temperature. Sections were washed with H_2_O and incubated in Nuclear Red 0,1% solution (MORPHISTO) for 5 min at room temperature. Paraffin sections were dehydrated in ascending series of 70% EtOH, 96% EtOH, 2‐propanol, and Xylol. Sections were mounted in Entellan and let dry overnight.

### Immunocytochemistry

Wholemount staining was performed in 48‐well plates. Fixed scaffolds were dissected in 2 mm^2^ pieces with a scalpel, and permeabilized in 0,2% Triton‐X in 1x PBS for 20 min. Samples were blocked for 25 min at RT, in 5% donkey serum diluted in antibody diluent solution. Primary antibodies were applied on a certain dilution overnight at 4 °C (CD45 1:200 (HI30, Invitrogen), vimentin 1:400 (Abcam, ab92547), MMP9 1:200 (Sigma, HPA001238)), LAMP1 1:400 (Cell Signaling Technologies, (D2D11) XP), *Salmonella* Typhimurium LPS 1:600 (Abcam, ab8274), GP2 1:200 (MBL, D277‐3)). Samples were washed in PBS‐T and incubated at RT for 1 h with secondary antibodies (donkey anti‐rabbit AF647, AF555 and donkey anti‐mouse AF647, AF555, or AF488) (Invitrogen). Samples were washed in PBS‐T and followed by 20 min incubation in 1:4000 or 1:6000 phalloidin (Abcam)/1:4000 DAPI (Thermo Scientific) at RT. Samples were washed in PBS‐T and mounted in Fluoromount G (Invitrogen).

### RNA‐FISH

For the detection of *SipB* RNA expression, probes were ordered and designed by Molecular Instruments. Samples were prepared as previously mentioned,^[^ ^14^ ^]^ with some modifications. Samples were fixed in 4% PFA for 2 h at room temperature. Samples were washed 2 times with 1x PBS^−^ (Sigma) and incubated in ice‐cold 70% ethanol at 4 °C overnight. Samples were washed with PBS and incubated in TEG buffer pH 7 with 25 µg mL^−1^ lysozyme for 10 min at room temperature. Samples were washed with 1x PBS‐T (1x PBS containing 0,05% Tween (Sigma)) and an HCR RNA‐FISH (Molecular Instruments) protocol was followed.

### Quantitative Real‐Time PCR

To isolate RNA, tissue models were washed with PBS and frozen directly at −80 °C. Following the manufacturer's instructions, RNA was isolated using the MicroSpin Total RNA kit (VWR). Synthesis of cDNA library was conducted with iScript cDNA Synthesis Kit (BioRad) according to manufacturer's instructions, in a thermal cycler (SensoQuest Labcycler Basic) programmed, 5 min at 25 °C, 30 min at 42 °C, 5 min at 85 °C and sustained at 12 °C. RT‐qPCR was performed in a C1000 Thermal Cycler (BioRad) with 25 ng cDNA using EvaGreen Supermix (Bio‐Rad) and a CFX 96 TouchTM Real‐Time PCR Detection 395 System (Bio‐Rad). List of primers is indicated on Table S2 (Supporting Information).

### Western Blot

Tissue models were disassembled and incubated in RIPA buffer containing protease inhibitor (Komplete, Roche) for 10 min on ice. Lysates were collected and stored at −80 °C until further analysis. Protein concentration was determined using the DC protein Assay Kit (Biorad). Absorbance was measured on a Tecan Infinite M200 multimode microplate reader and the desired amount of protein was undergone chloroform‐methanol precipitation. Electrophoresis was performed on an 8% and 6% poly‐acrylamide SDS‐PAGE. Samples of 25 µg were loaded per well and let run on a PerfectBlue Twin S (Peqlab) for 50–60 min at 25 mA. For villin‐1 semi‐dry transfer was conducted on a PerfectBlue semi‐dry Blotter (Peqlab). For MUC‐2, wet transfer was performed on a VWR PerfectBlue TANK Electric Blotter Web S (VWR) overnight at 4 °C. Nitrocellulose membranes were blocked for 1 h at RT in 1x TBST with 5% milk powder (Roth). Villin‐1 and MUC‐2 antibodies were diluted 1:1000 in TBST with 5% milk powder and membranes were incubated overnight at 4 °C. Membranes were then washed in TBST and incubated with HRP‐goat anti‐rabbit (Johnson ImmunoResearch) diluted 1:10 000 in TBST with 5% milk for 1 h. Detection was performed using the WesternBright Chemiluminescence Substrate Quantum kit (Biozym). Images were acquired in a ChemiDoc Imaging System (Biorad).

### Inflammatory Cytokine Measurement

Inflammatory cytokines were detected in supernatants collected from the basolateral compartment of the models using the BD Cytometric Bead Array (CBA) Human Inflammatory Cytokine Cytometric Bead Array kit (BD) according to manufacturer's instructions. Measurement was performed on a BD Accuri C6 Plus with an integrated BD Sampler Plus, robotic arm system (BD). Inflammatory cytokine data were analyzed with the FCAP software (BD).

### Transmission Electron Microscopy (TEM)

Preparation of samples for electron microscopy was conducted according to the previously published protocol.^[^ ^74^ ^]^ Transmission electron microscopy was performed at 120 kV acceleration voltage at a JEM‐1400 Flash (JEOL, Germany) transmission electron microscope equipped with a Matataki 2k x 2k camera.

### Scanning Electron Microscopy (SEM)

For scanning electron microscopy samples were prepared as previously described.^[^ ^75^ ^]^ Imaging was conducted on JEOL JSM‐7500F field emission scanning electron microscope.

### Light Microscopy

Epifluorescence images were acquired on a Keyence BX‐800. Confocal imaging was conducted on a Leica SP‐8 Confocal Laser Scanning microscope. For live cell imaging, flattened model inserts were mounted on 24‐well glass‐bottom plates using a Matrigel droplet as glue. Upon cross‐linking, plates were transferred to a Leica SP‐8 with a 37 °C pre‐warmed chamber. Imaging was conducted using an HC APO L 20x/0,50 U‐V‐I.

### CFU Assay

For the CFU assay, supernatants were collected from the apical compartment of the models. Serial dilutions of 10^−3^ – 10^−7^ were performed and 10 µL droplets were plated on LB agar with chloramphenicol. Plates were incubated for 8 h at 37 °C or 14–16 h at 30 °C. Colonies were counted and the CFU/100 µL, representing the actual number of STm on the apical compartment, was calculated using:

(1) CFU/100μL=m×D×10

*m* = mean of colonies counted, *D* = dilution factor.

### Lactate Dehydrogenase (LDH) Assay

Lactate dehydrogenase release was detected in supernatants collected from the basolateral compartment using the CyQUANT LDH Cytotoxicity Assay Kit according to manufacturer's instructions. Absorbance was measured on a Tecan reader.

### Image Analysis

Images were processed and analyzed with Fiji (ImageJ 2.9.0). Quantification for MMP9 percentage and infection rate was conducted with manual counting on at least 3 different fields. CD45 positive cells with attached or intracellular *Salmonella* were considered as infected. For LAMP1 and STmLPS co‐localization the Fiji plugin DiAna was implemented. Luminal area percentage was estimated for STmLPS and *sipB* mRNA by converting the epithelial layer to an XY 3D projection and the area was measured. 3D live cell imaging movies were generated within Imaris (Oxford Instruments).

### RNA‐Seq

RNA was isolated using the MicroSpin Total RNA kit (VWR). RNA quality was checked using a 5200 Fragment Analyzer with the DNF‐471‐33 – SS Total RNA 15 nt kit (Agilent Technologies). cDNA libraries suitable for sequencing were prepared from 100 ng of total RNA with oligo‐dT capture beads for polyA‐based mRNA enrichment using the TruSeq Stranded mRNA Library Preparation Kit (Illumina) according to manufacturer's instructions (yet scaled down to ½ volume). After 15 cycles of PCR amplification, the size distribution of the barcoded cDNA libraries was estimated to be ≈337 bp by electrophoresis on 5200 Fragment Analyzer with DNF‐474‐33 – HS NGS Fragment 1–6000 bp kit.

Sequencing of pooled libraries spiked with 1% PhiX control library, was performed at 25 million reads per sample in single‐end mode with 100 nt read length on the NextSeq 2000 platform (Illumina) using a P3 sequencing kit. Demultiplexed FASTQ files were generated with bcl2fastq2 v2.20.0.422 (Illumina).

In total, four culture conditions (epithelium (E), epithelium‐fibroblast (EF), epithelium‐MDM (EM), and epithelium‐MDM‐fibroblast (EMF)), obtained from three donors, were analyzed. Quality control of the raw RNA sequencing data was performed using FastQC (version 0.11.9)^[^ ^76^ ^]^ and MultiQC (version 1.12)^[^ ^77^ ^]^ for summarizing the FastQC results.

### Hardware and Software

Quality control and alignment as well as all R‐based bioinformatics analyses were performed in a virtual Ubuntu environment (version 22.04.4 LTS, Jammy Jellyfish) using conda (version 22.11.1)^[^ ^78^ ^]^ for package and environment management. The virtual machine operated on a host system with the Linux kernel 6.5.0‐44‐generic, running on a computer with with an AMD Ryzen 9 3900X, 12‐core processor, and 64 GB RAM. Data analyses were performed in R (version 4.0.5)^[^ ^79^ ^]^ using RStudio (2023.03.0 + 386 “Cherry Blossom”).

### Data Preprocessing

The raw reads were aligned to the human genome reference (GRCh38, primary assembly) GENCODE v46 (GRCh38.p14),^[^ ^80^, ^81^ ^]^ which corresponds to Ensembl^[^ ^82^ ^]^ version 112, using STAR (version 2.7.10a).^[^ ^83^ ^]^ Alignment was performed in two‐pass mode to enhance mapping accuracy.

Gene expression quantification was performed using RSEM (version 1.3.1).^[^ ^84^ ^]^ The reference preparation involved the GENCODE v46 annotation aligned to the GRCh38 primary assembly genome. The RSEM quantification was executed on BAM aligned to the transcriptome files generated from the STAR alignment.

### Data Analysis: Identification and Visualization of Differentially Expressed Genes

Differential expression analysis was conducted in R (version 4.0.5) using the DESeq2 package (version 1.44.0)^[^ ^85^ ^]^ combined with tximport (version 1.32.0)^[^ ^86^ ^]^ for data import. Variance‐stabilizing transformation (VST) was applied to the count data to facilitate Principal Component Analysis (PCA) and clustering. Log fold change shrinkage was performed using the apeglm package (version 1.26.1).^[^ ^87^ ^]^ For our analyses, all genes with an adjusted *p*‐value of < 0.05 and an absolute log2 fold change of > 1 (>1 for upregulated genes and < −1 for downregulated genes) were considered as DEGs. The following analyses were performed for all possible comparisons (EMF v E, EF vs E, EM vs E, EMF vs EF, EM vs EF, EMF vs EM), results not shown are available in Tables S3–S8 (Supporting Information).

### Data Analysis: Enrichment Analyses

Subsequently, Gene Set Enrichment Analysis (GSEA) as well as Gene ontology (GO) pathway enrichment analyses and KEGG enrichment analysis were performed using ClusterProfiler (version 4.12.0),^[^ ^88^, ^89^ ^]^ enrichplot (version 1.24.0)^[^ ^90^ ^]^ and org.Hs.eg.db (version 3.19.1).^[^ ^91^ ^]^ Annotated gene sets for enrichment analyses were provided via various databases, including org.Hs.eg.db,^[^ ^91^ ^]^ the Molecular Signatures Database (MSigDB),^[^ ^92^, ^93^ ^]^ and the Kyoto Encyclopedia of Genes and Genomes (KEGG).^[^ ^94^, ^95^ ^]^

### Data Visualization

Visualization of the data was performed using various R packages. The differences in gene expression were visualized as Principal Feature Analysis (PCA) using DESeq2 (version 1.44.0).^[^ ^85^ ^]^ The PCA plots were generated with ggplot2 (version 3.5.1).^[^ ^96^ ^]^ Heatmaps were produced with the pheatmap package (version 1.0.12),^[^ ^97^ ^]^ and as volcano plots with EnhancedVolcano (version 1.22.0). Enrichment results were displayed using dot plots for GSEA and bar plots for KEGG pathway enrichment with enrichplot (version 1.24.0).^[^ ^90^ ^]^ All plots were customized using the Cairo package (version 1.6.2)^[^ ^98^ ^]^ and the extra font package (version 0.19).^[^ ^99^ ^]^ Additional packages used during data analysis or for creating the plots and figures include tidyverse (version 2.0.0),^[^ ^100^ ^]^ biomaRt (version 2.60.1).^[^ ^101^ ^]^

### Cell Type Deconvolution

The R‐package MuSiC^[^ ^102^ ^]^ performs Multi‐subject Single‐cell Deconvolution, which allows the estimation of cell type proportions from bulk sequencing data.^[^ ^102^ ^]^ Its extension, MuSiC2,^[^ ^103^ ^]^ allows performing deconvolution analyses of bulk RNA‐sequencing data with multiple clinical conditions, with at least one condition being different from the single‐cell reference.^[^ ^103^ ^]^ This is especially useful due to the challenges of obtaining single‐cell sequencing data for diseased samples, which are rare but often required as references for bulk RNA‐sequencing data deconvolution methods.^[^ ^103^ ^]^

The cell‐type specific gene expressions were extracted from single‐cell RNA sequencing (RNA‐seq) data and subsequently used to identify the composition of various cell types within bulk RNA‐seq data.^[^ ^102^ ^]^ The bulk RNA‐seq data was prepared as described before and the resulting RSEM files were imported in R using tximport^[^ ^86^ ^]^ (version 1.32.0). The count data was subsequently annotated with gene names using the biomaRt^[^ ^101^ ^]^ database (version 2.60.1) and Ensembl^[^ ^82^, ^104^ ^]^ version 112, which corresponds to the GENCODE^[^ ^80^, ^81^ ^]^ version used for alignment). Cells containing empty gene names or duplicate gene names were removed, and the annotated counts data was converted into a matrix, which was, together with the meta data information about the different samples, organized into an ExpressionSet using Biobase^[^ ^105^ ^]^ (version 2.64.0), and saved as an RDS file.

For analysis, data provided were used via the Gut Cell Atlas, a publicly available single‐cell dataset of the human gut,^[^ ^106^ ^]^ which is part of the Human Cell Atlas and available at https://www.gutcellatlas.org/. The raw data, which is available as an H5AD file, was converted back into raw format mimicking 10x output in Python (version 3.9.19) by using scanpy^[^ ^107^ ^]^ (version 1.10.2) and scipy^[^ ^108^ ^]^ (version 1.13.1) was filtered to include only “Healthy adult” samples from the “SmallInt” region. The resulting files were imported into R using the Read10x function of Seurat^[^ ^109^, ^110^, ^111^, ^112^, ^113^ ^]^ (version 5.1.0). The processed data was subsequently converted to a SingleCellExperiment object via SingleCellExperiment^[^ ^114^ ^]^ (version 1.26.0) and saved as RDS file. The desired cell types were defined as Enterocyte, BEST4+ epithelial, and Goblet cell. Their presence in the single‐cell data was verified. Additionally, the cell type annotations of the SingleCellExperiment object (Single Cell Experiment^[^ ^114^ ^]^ version 1.26.0) were standardized for TOAST^[^ ^115^, ^116^, ^117^, ^118^ ^]^ analysis by replacing spaces and special characters with underscores.

Deconvolution was performed using MuSiC2^[^ ^103^ ^]^ (version 0.1.0), which uses functions of the MuSiC^[^ ^102^ ^]^ package (version 1.0.0), in conjunction with TOAST,^[^ ^115^, ^116^, ^117^, ^118^ ^]^ (version 1.18.0) using bulk RNA‐sequencing data of epithelial cells (E cells, prepared as ExpressionSet, as described above) as “control” data and epithelial, mesenchymal, fibroblast (EMF, prepared as ExpressionSet, as described above) cells as “case” samples. Both, E cells and EMF cells were acquired in ExpressionSet format and loaded via the readRDS function of Biobase^[^ ^105^ ^]^ (version 2.64.0), and subsequently transformed into matrix format for downstream analysis. Parameters were set to perform 20 resampling iterations with a 50% sampling proportion, and cutoff thresholds for convergence and residuals were set at 0,05 and 0,01, respectively.

Subsequently, TOAST (TOol for Analyzing STatistical significance)^[^ ^115^, ^116^, ^117^, ^118^ ^]^ (version 1.18.0) was employed to assess the statistical significance of the deconvolution results obtained from MuSiC2.

To visualize cell type proportions across control and case groups, violin plots were generated using ggplot2^[^ ^96^ ^]^ (version 3.5.1). These plots included statistical significance annotations. For each cell type, a t‐test was performed to compare proportions between control and case groups, and the resulting *p*‐values were adjusted for multiple comparisons using the Benjamini‐Hochberg method. The ggsignif package^[^ ^119^ ^]^ (version 0.6.4) was used to add significance annotations to the violin plots, and the extrafont package^[^ ^99^ ^]^ (version 0.19) was used for customized text elements.

The combined data for plotting included cell type proportions, sample IDs, and cell type annotations. The data were scaled and cleaned of NA/NaN/Inf values. For each cell type, a t‐test was performed if the group factor had exactly two levels, comparing the proportions between control and case groups. The significance level was indicated on the plots as ‚ (***) for *p* ≤ 0001, (**) for *p* ≤ 0,01, (*) for *p* ≤ 0,05, and “ns” for not significant.

Additional packages used for the analyses were hdf5r^[^ ^120^ ^]^ (version 1.3.11), rhdf5^[^ ^121^ ^]^ (version 2.48.0), reticulate^[^ ^122^ ^]^ (version 1.38.0), SeuratDisk^[^ ^123^ ^]^ (version 0.0.0.9021), tidyverse^[^ ^100^ ^]^ (version 2.0.0), reshape2^[^ ^124^ ^]^ (version 1.4.4) cowplot^[^ ^125^ ^]^ (version 1.1.3), Cairo^[^ ^98^ ^]^ (version 1.6‐2), and pheatmap^[^ ^97^ ^]^ (version 1.0.12).

### Statistical Analysis

Data were analyzed and graphs were generated using MATLAB R2021a (Mathworks). Comparative analysis was conducted with two‐tailed Student's *t*‐test, Wilcoxon‐Mann‐Whitney (U‐test), Friedman's or Kruskal‐Wallis test with Dunn's post‐hoc test and N‐way ANOVA with Tukey's post‐hoc test. Values of *p* ≤ 0,05 were considered significant.

## Conflict of interest

The authors declare no conflict of interest.

## Supporting information



Supporting Information

Supplemental Video 1

Supplemental Table 1

Supplemental Table 2

Supplemental Table 3

Supplemental Table 4

Supplemental Table 5

Supplemental Table 6

Supplemental Table 7

Supplemental Table 8

## Data Availability

The data that support the findings of this study are available in the supplementary material of this article.
