# Supplementary material for: A Multicellular In Vitro Model of the Human Intestine with Immunocompetent Features Highlights Host‐Pathogen Interactions During Early Salmonella Typhimurium Infection
Source: Adv Sci (Weinh). 2025 Jan 14;12(9):2411233. doi: 10.1002/advs.202411233 (PMC11884561; doi:10.1002/advs.202411233)
Supplement: Supplementary file 1 — Supporting Information [file ADVS-12-2411233-s001.docx]

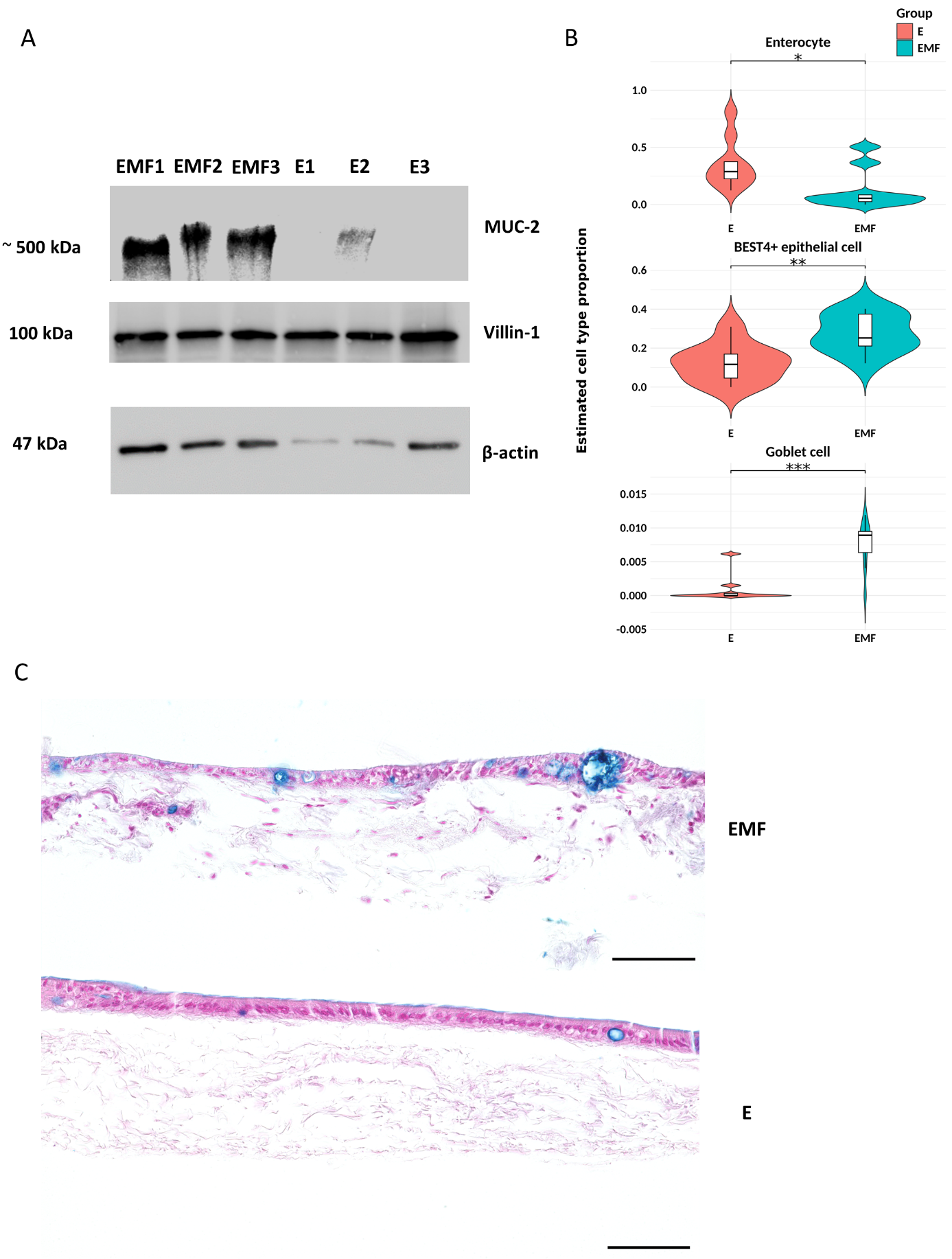


**Figure S1** (A) Western blot analysis shows the protein expression of Villin-1 and MUC-2 in each three replicates of monoculture and co-culture models. Beta-actin was the loading control. (B) Deconvolution of bulk RNA-seq data comparing the cell-type frequency for enterocytes, BEST4-positive epithelial cells and Goblet cells between E and EMF models. (*) *p* ≤ 0.05, (**) *p* ≤ 0.01, (***) *p* ≤ 0.001. (C) Alcian blue-stained, paraffin-embedded epithelial models, with or without macrophages and fibroblasts. Scale bar= 100 µm. MUC-2: mucin-2, BEST4: Bestrophin-4, E: epithelium only, EMF: epithelium-MDM-fibroblast model.


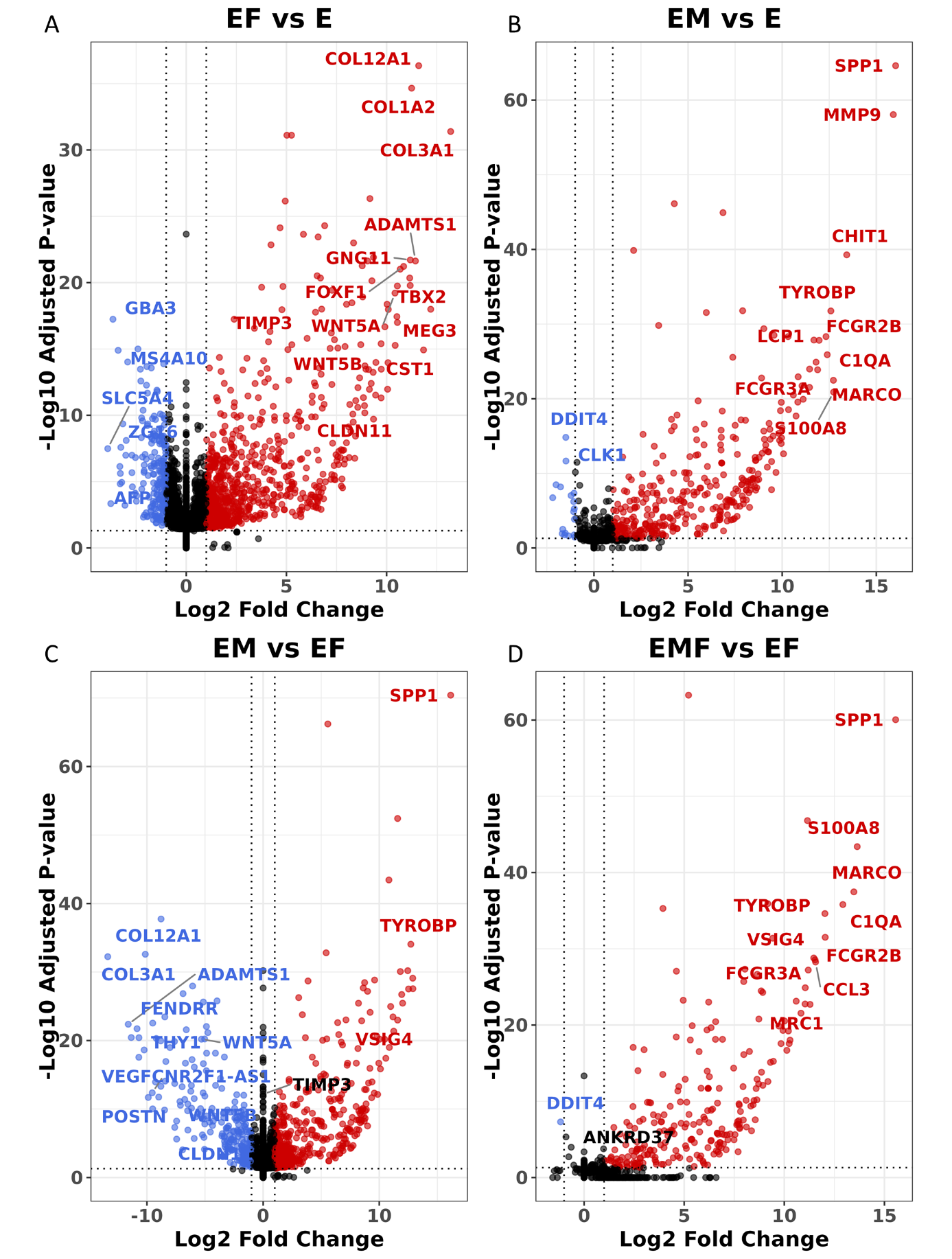


**Figure S2** (A-D) Volcano plots showing differentially expressed genes between the indicated culture conditions as inferred from RNA-seq analysis. E= epithelium, EF = epithelium-fibroblasts, EM = epithelium-MDMs, EMF = epithelium-MDMs –fibroblasts.


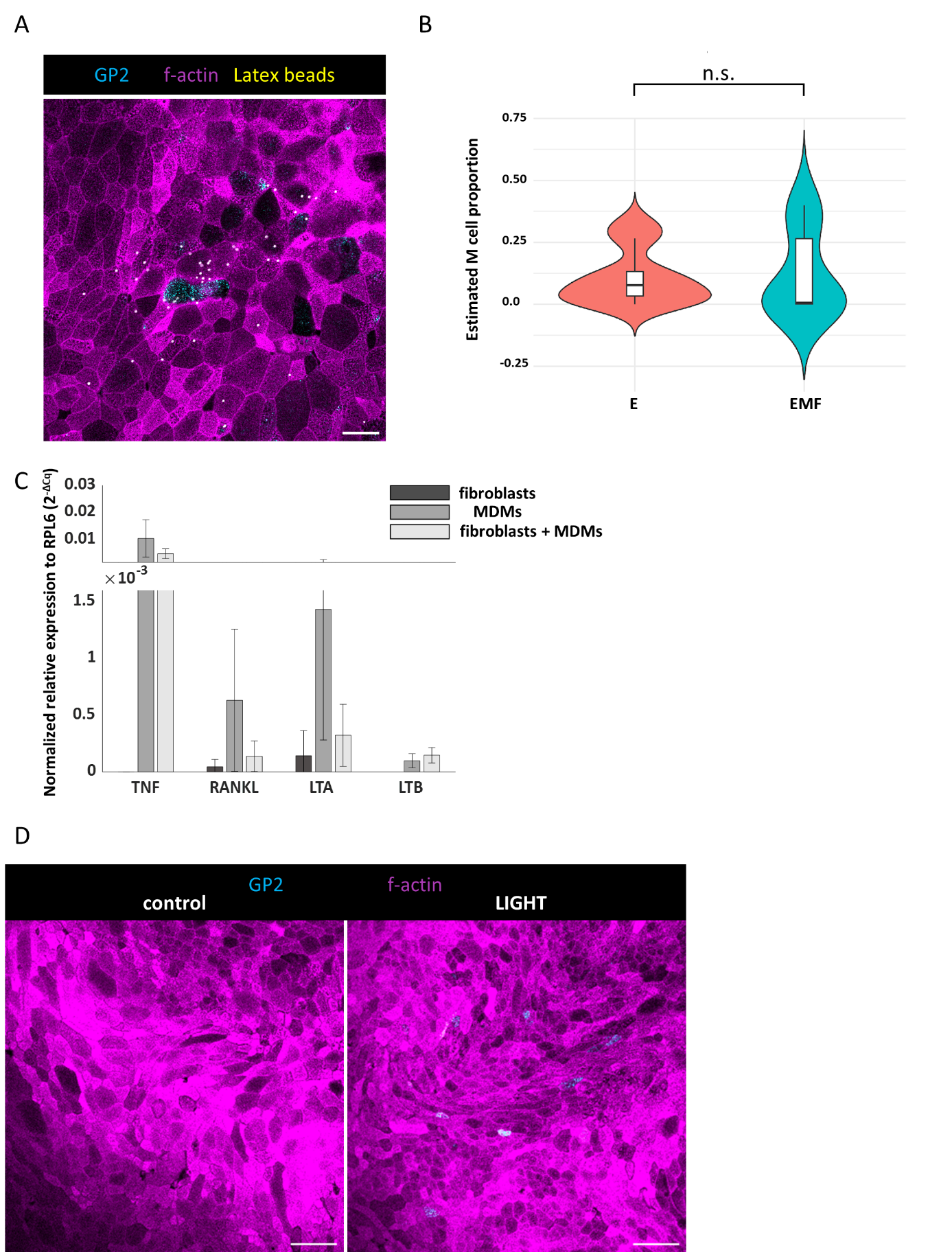


**Figure S3** (A) Uptake of latex beads by GP2-positive cells. (n=2). (B) Violin plot shows the estimated M-cell proportion after deconvolution of the bulk RNA-seq data, comparing epithelial monocultures (E) to the multicellular model (EMF). n.s.: non-significant. (C) qRT-PCR analysis of *TNFRL* expression in mesenchymal cells. Bars and error bars represent the mean and standard error over three replicates. (D) Confocal images show enteroid monocultures differentiated in presence of different TNFRLs. GP2 is shown in cyan and F-actin in magenta. Scale bar = 40 µm, (n=3). GP2: Glycoprotein-2, TNF: Tumor Necrosis Factor, RANKL: Receptor Activator Of Nuclear Factor Kappa B Ligand, LTA: Lymphotoxin Alpha, LTB: Lymphotoxin Beta, E: epithelium, EMF: epithelium-MDM-fibroblast.


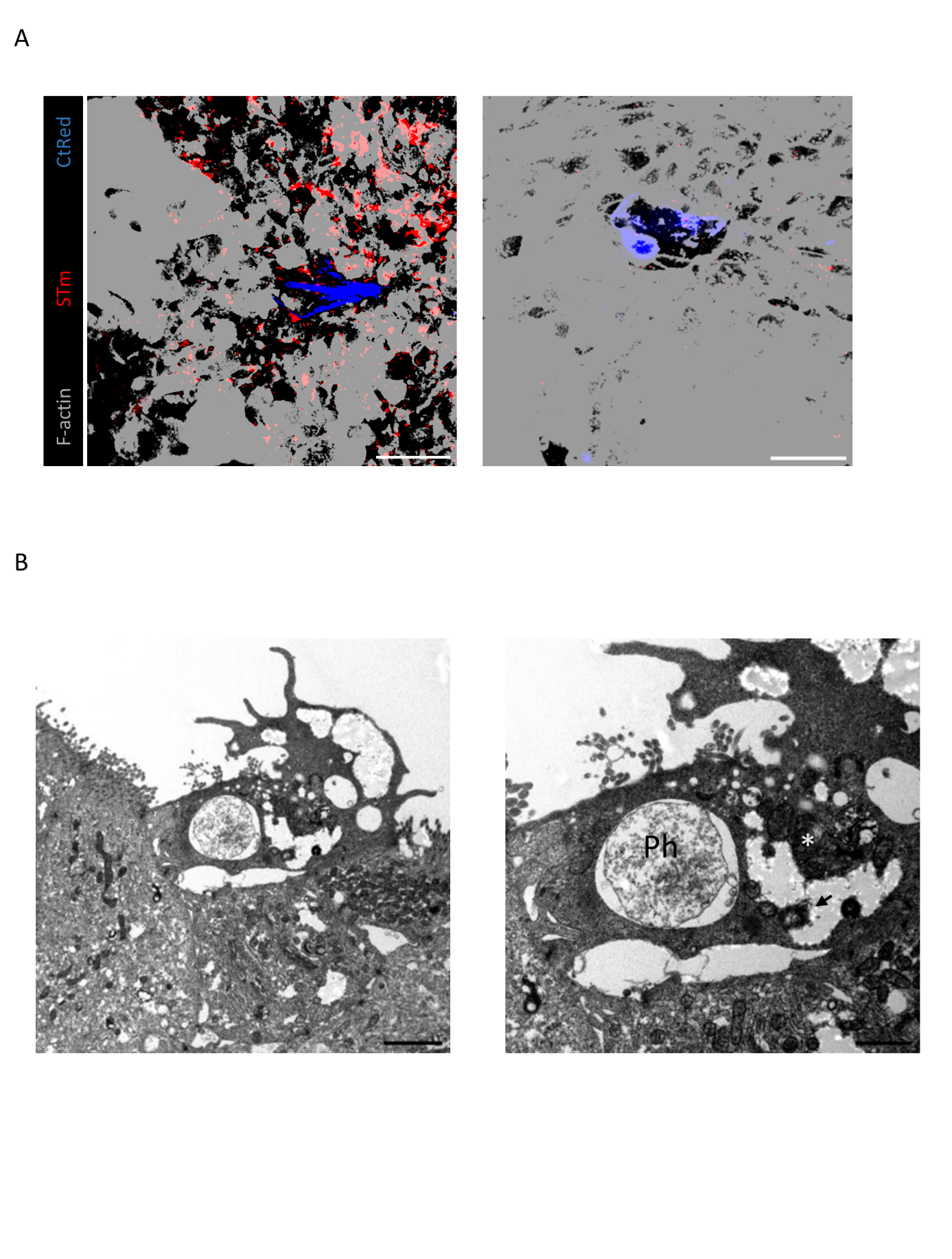


**Figure S4** (A) Fluorescence confocal images of MDMs located at the luminal side of the EMF model. Scale bars = 40 µm. (n=1) (B) TEM images showing a macrophage in the luminal compartment, (n=1). Scale bars = 2 µm (low magnification), 1 µm (high magnification). (Ph) phagosome, (*) nucleus, (arrowhead) vacuolar STm (n=1). CtRed: CellTracker^TM^.


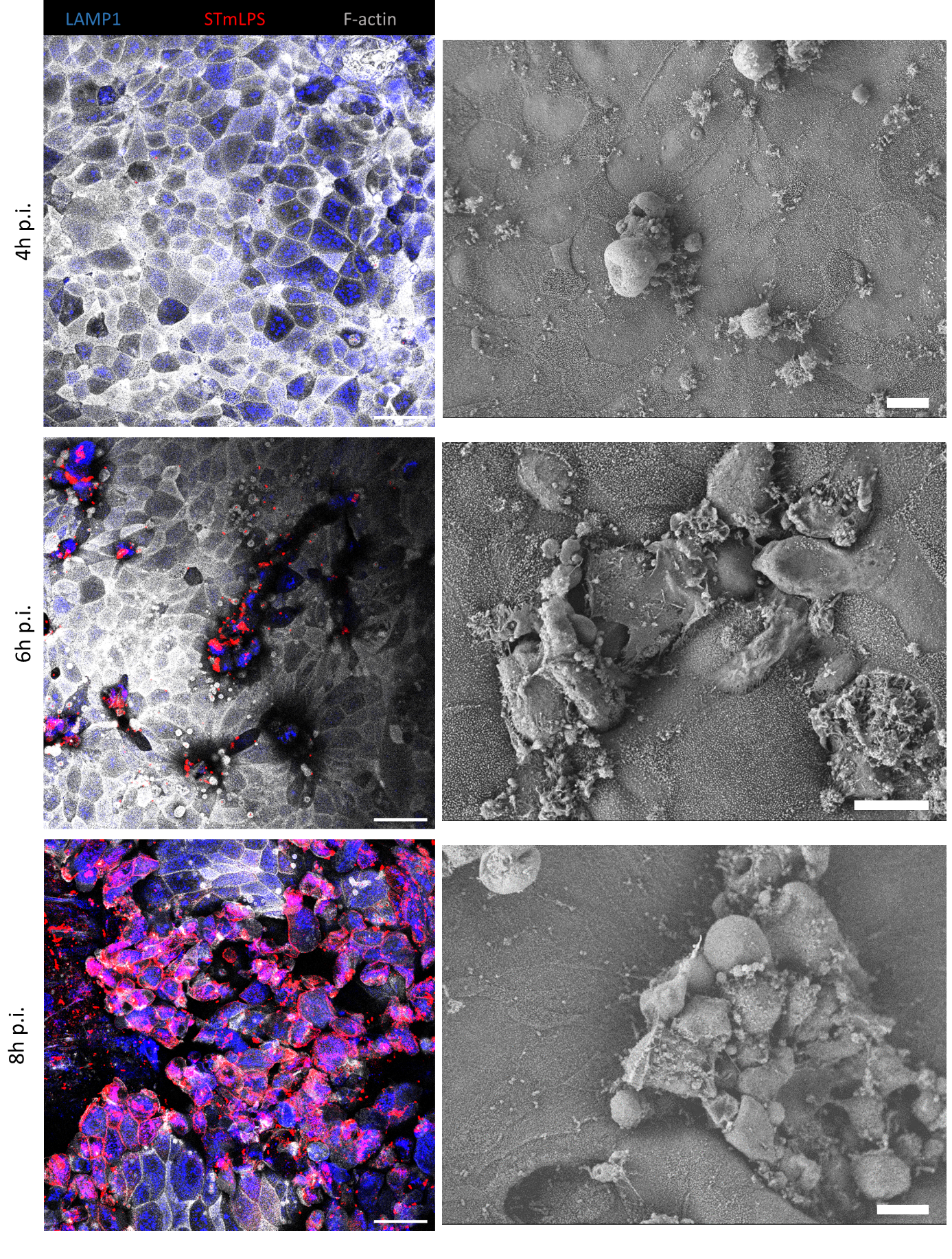


**Figure S5** Confocal microscopy (left) and scanning electron microscopy (right), showing the extrusion of intestinal epithelial cells during STm infection. Confocal images: scale bars = 40 µm. (n=3). Scanning electron microscopy images: scale bars = 10 µm. (n=1). LAMP1: Lysosomal-Associated Membrane Protein 1, STmLPS: *S*. Typhimurium lipopolysaccharide.
